# The Reactivity, Distribution and Abundance of Non-Astrocytic Inner Retinal Glial (NIRG) Cells Are Regulated by Microglia, Acute Damage, and IGF1

**DOI:** 10.1371/journal.pone.0044477

**Published:** 2012-09-04

**Authors:** Christopher P. Zelinka, Melissa A. Scott, Leo Volkov, Andy J. Fischer

**Affiliations:** Department of Neuroscience, College of Medicine, The Ohio State University, Columbus, Ohio, United States of America; Zhejiang University School of Medicine, China

## Abstract

Recent studies have described a novel type of glial cell that is scattered across the inner layers of the avian retina and possibly the retinas of primates. These cells have been termed Non-astrocytic Inner Retinal Glial (NIRG) cells. These cells are stimulated by insulin-like growth factor 1 (IGF1) to proliferate, migrate distally into the retina, and become reactive. These changes in glial activity correlate with increased susceptibility of retinal neurons and Müller glia to excitotoxic damage. The purpose of this study was to further study the NIRG cells in retinas treated with IGF1 or acute damage. In response to IGF1, the reactivity, proliferation and migration of NIRG cells persists through 3 days after treatment. At 7 days after treatment, the numbers and distribution of NIRG cells returns to normal, suggesting that homeostatic mechanisms are in place within the retina to maintain the numbers and distribution of these glial cells. By comparison, IGF1-induced microglial reactivity persists for at least 7 days after treatment. In damaged retinas, we find a transient accumulation of NIRG cells, which parallels the accumulation of reactive microglia, suggesting that the reactivity of NIRG cells and microglia are linked. When the microglia are selectively ablated by the combination of interleukin 6 and clodronate-liposomes, the NIRG cells down-regulate transitin and perish within the following week, suggesting that the survival and phenotype of NIRG cells are somehow linked to the microglia. We conclude that the abundance, reactivity and retinal distribution of NIRG cells can be dynamic, are regulated by homoestatic mechanisms and are tethered to the microglia.

## Introduction

The retinas of vertebrates contain many different types of glial cells. Consistent across all vertebrate species, retinal glia include Müller glia - derived from retinal stem cells [1], and microglia - derived from yolk sac stem cells [2], [3]. With significant variations between species, retinal glia can include astrocytes and oligodendrocytes. For example, the retinas of chickens, guinea pigs and rabbits contain oligodendrocytes that myelinate the axons of ganglion cells in the nerve fiber layer (NFL) [4], [5], [6]. By comparison, the retinas of guinea pigs and birds do not appear to contain conventional types of astrocytes [7], [8], [9], [10]. In addition to the well-described conventional types of retinal glia, recent reports have described a novel type of glial cell scattered across inner layers of the chick retina [8], [11]. We termed these cells Non-astrocytic Inner Retinal Glia-like (NIRG) cells. Rompani and Cepko (2010) described “diacytes” and astrocytes that are likely to be the same cells that we described as NIRG cells [11]. The NIRG cells (also known as diacytes/astrocytes) are derived from multipotent progenitors in the optic stalk that also give rise to optic nerve astrocytes and oligodendrocytes [11]. However, the Pax2-expressing optic nerve glial progenitors are never observed within the retina [12], [13]. We reported that the NIRG cells have a unique, distinct phenotype and can be stimulated by intraocular injections of IGF1 [8]. We found that the IGF1 receptor was expressed by cells, likely NIRG cells and/or microglia, scattered across the inner retinal layers, but not by cells in the inner and outer nuclear layers. The NIRG cells express vimentin and transitin (the avian homologue of nestin), similar to Müller glia and retinal progenitors. In addition, the NIRG cells express the transcription factors Sox2, Sox9, Nkx2.2 [8]. However, these cells do not express significant levels of well-established markers for astrocytes and Müller glia such as S100β, GFAP (Glial Fibrilliary Acidic Protein), Top_AP_ or glutamine synthetase. Further, the NIRG cells do not up-regulate GFAP in response to acute damage [8], nor do they express Pax2, unlike to the optic nerve astrocytes in the chick and the astrocytes in the retinas of mice, dogs and primates [12]. The NIRG cells are distinct from retinal microglia in that they are negative for CD45, RCA1 and lysosomal membrane glycoprotein [8]. The NIRG cells are distinct from retinal oligodendrocytes in that they are negative for transferrin-binding protein [8], proteolipid protein, myelin/oligodendrocytes-specific protein, and myelin-associated glycoprotein [11]. The NIRG cells are not present in the retinas mice and guinea pigs, whereas NIRG-like cells were found in the retinas of dogs and non-human primates [14].

**Figure 1:**
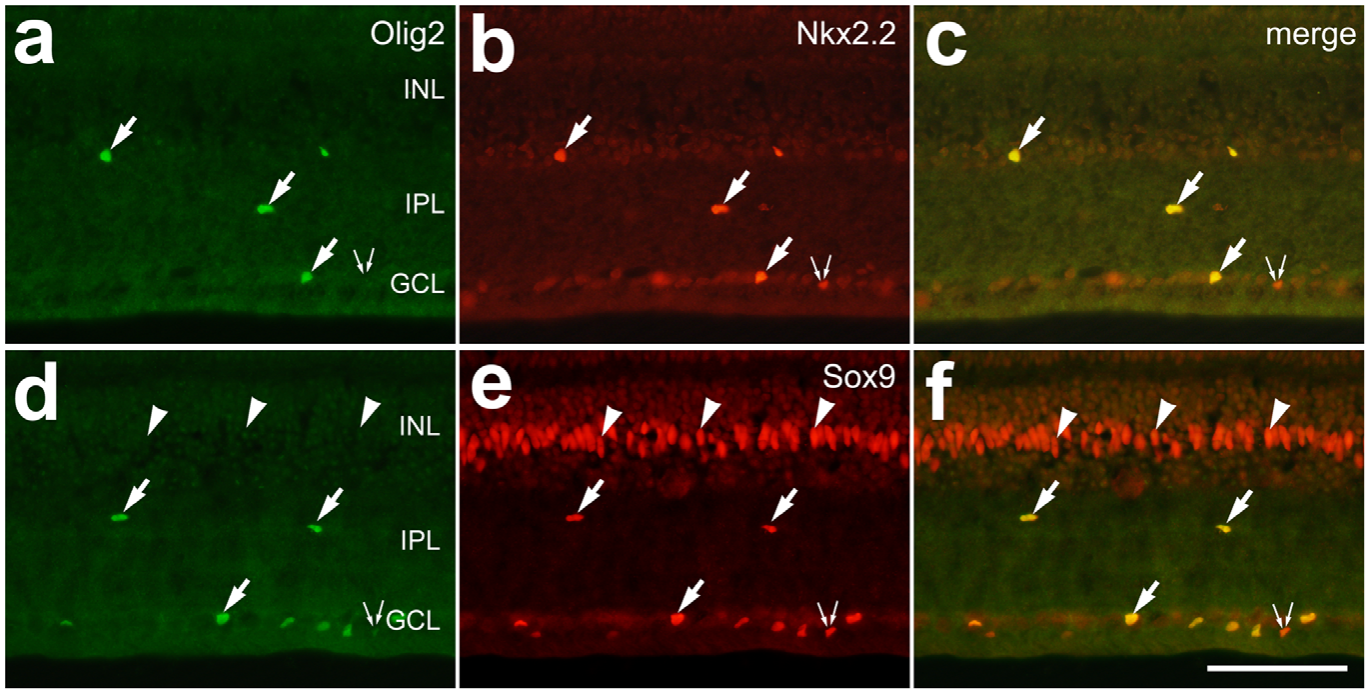
Olig2 is expressed by Nkx2.2/Sox9-positive NIRG cells. Vertical sections of the retina were labeled with antibodies to Oilg2 (green) and Nkx2.2 (red) (**a**–**c**) or Olig2 (green) and Sox9 (red) (**d**–**f**). Arrows indicate the nuclei of cells labeled for Olig2 and Nkx2.2 or Olig2 and Sox9, arrow-heads indicate the nuclei of Sox9-positive Müller glia, and small double-arrows indicate cells in the GCL that are labeled for Sox9 or Nkx2.2 alone. The scale bar (50 µm) in panel **f** applies to **a**–**f**. Abbreviations: INL – inner nuclear layer, IPL – inner plexiform layer, GCL – ganglion cell layer.

The functions of the NIRG cells within the retina remain uncertain. IGF1 stimulates retinal glia: (i) the NIRG cells proliferate, migrate distally into the retina, and up-regulate transitin, (ii) the microglia up-regulate CD45 and acquire ameboid morphology, and (iii) Müller glial accumulate p38 MAPK and cFos [8]. With Müller glia, microglia and NIRG cells stimulated by IGF1, there were elevated levels of cell death and wide-spread focal retinal detachments in response to an excitotoxic insult [8]. The increased cell death was prominent within areas of retinal detachment which were coincident with a stark loss of Müller glia and an accumulation of NIRG cells [8]. Many questions remain unresolved regarding the nature of the NIRG cells and their responses to IGF1 and retinal damage. Therefore, the purpose of this study was to better characterize the NIRG cells in retinas treated with IGF1, acute damage, or when the microglia have been selectively ablated.

**Figure 2:**
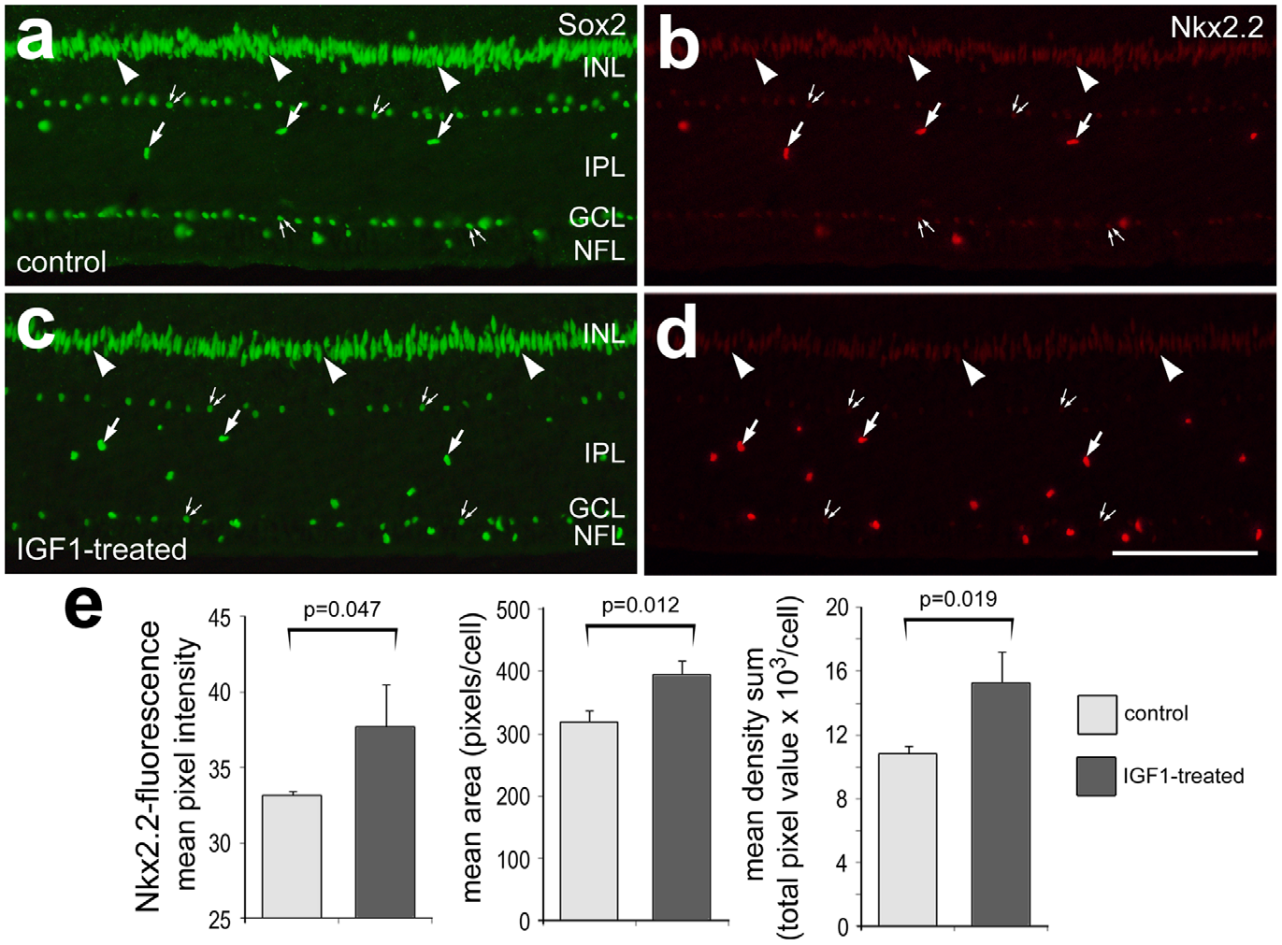
IGF1 stimulates NIRG cells to up-regulate Nkx2.2, but not Sox2. Retinas were obtained from eyes that received 2 consecutive daily injections vehicle (control) or IGF1 (treated). Vertical sections of the retina were labeled with antibodies to Sox2 (green; **a** and **b**) and Nkx2.2 (red; **c** and **d**). Images of control and treated retinas were obtained using identical microscope, camera and post-acquisition processing settings. Arrows indicate the nuclei of NIRG cells that are labeled for Sox2 and Nkx2.2, arrow-head indicate nuclei of Müller glia, and small double-arrows indicate the nuclei of cholinergic amacrine cells. The histograms in panel **e** illustrate the mean (±SD) pixel intensity, area and density sum for Nkx2.2-immunofluorescence per cells. Image Pro 6.2 was used to measure total area for pixel intensities >68 (0 = black, 255 = saturated green), average pixel intensity and the density sum, as described in the Methods. Immunofluorescence for Nkx2.2 was measured in individual nuclei for ≥50 cells per individual, per experimental condition. Significance of difference between control and IGF1-treated retinas was determined by using a two-tailed Student’s t-test. The scale bar (50 µm) in panel **d** applies to **a**–**d**. Abbreviations: ONL – outer nuclear layer, INL – inner nuclear layer, IPL – inner plexiform layer, GCL – ganglion cell layer.

**Figure 3:**
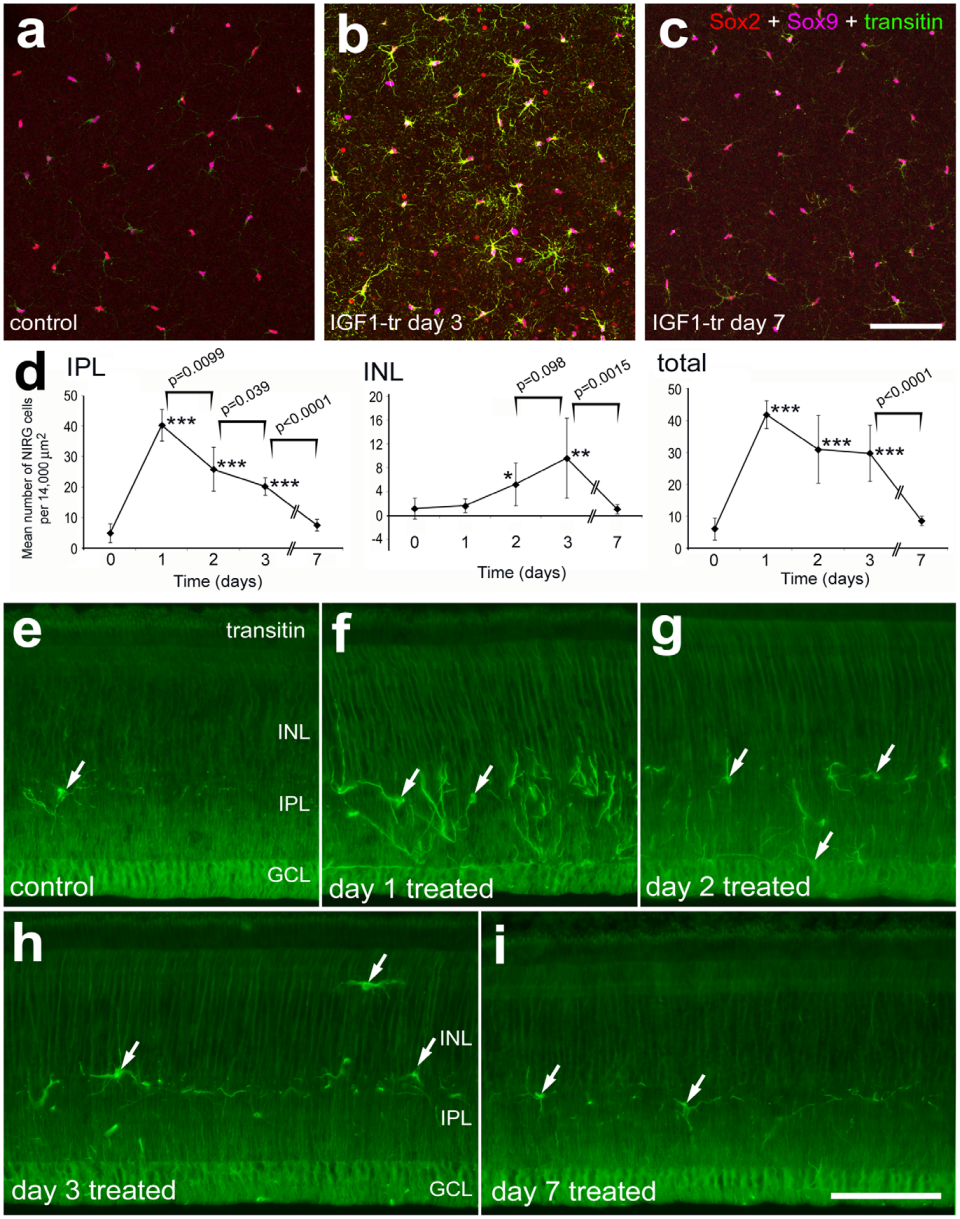
IGF1 causes a transient reactive phenotype and accumulation of NIRG cells in the retina. Whole-mount preparations of control and treated retinas were labeled with antibodies to Sox2 (red), Sox9 (magenta), and transitin (green) (**a**–**c**). Vertical sections of the retina were labeled with antibodies to transitin (**e**–**i**). Micrographs were obtained from projections of optical sections collected through the IPL (**a**–**c**) or wide-field microscopy of retinal sections (**e**–**i**). Images of control and treated retinas were obtained using identical microscope, camera settings, and post-acquisition processing settings. Histograms illustrate the mean (± SD) number of NIRG cells within the IPL, INL and the entire retina (**d**). Significance of difference among groups was determined by using a one-way ANOVA (p<0.0001). Significance of difference between control (time 0) and treatment groups (days 1, 2, 3 and 7 after IGF1-treatment) (*p<0.05, **p<0.001, ***p<0.0001), and between treatment groups at different time points (bracket and p-values) was determined by using a two-tailed post-hoc Student’s t-test. The scale bar (50 µm) in panel **c** applies to **a**–**c**, and the panel in **i** applies to **e**–**i**. Abbreviations: INL – inner nuclear layer, IPL – inner plexiform layer, GCL – ganglion cell layer.

## Results

### The NIRG Cells Express Olig2

A recent report by Rompani and Cepko (2010) described glial cells, putative astrocytes and newly identified “diacytes”, in the IPL and ganglion cell layer (GCL) of the chick retina [11]. These glial cells are derived from progenitors in the developing optic nerve and express the bHLH transcription factor Olig2 [11]. We believe that the NIRG cells are the same cells as those described by Rompani and Cepko as the astrocytes and diacytes. To test this hypothesis, we examined whether Olig2 was expressed by NIRG cells that are positive for Nkx2.2 and Sox9. All of the NIRG cells within the IPL express Sox2, Sox9, Nkx2.2 and transitin, whereas [8]. We found that all of the Olig2-positive cells (n = 389) that are scattered across the IPL, GCL and nerve fiber layer (NFL) were positive for Nkx2.2 (Figs. 1a–c). However, not all of the Nkx2.2-positive cells were positive for Olig2; about 5% (22 of 411 cells) of the Nkx2.2-positive cells were negative for Olig2 and all of these cells were found in the GCL (Figs. 1a–c). Similarly, all of the Olig2-positive cells (n = 426) were positive for Sox9, whereas a minority (25 of 451) of the Sox9-positive cells were Olig2-negative and all of these cells were found in the GCL (Figs. 1d–f). The Sox9+/Nkx2.2+/Olig2- and some of the Sox9+/Nkx2.2+/Olig2+ cells in the GCL and NFL were oligodendrocytes (data not shown), consistent with our previous findings that oligodendrocytes in the GCL and NFL express Sox9 and/or Nkx2.2 [8].

**Figure 4:**
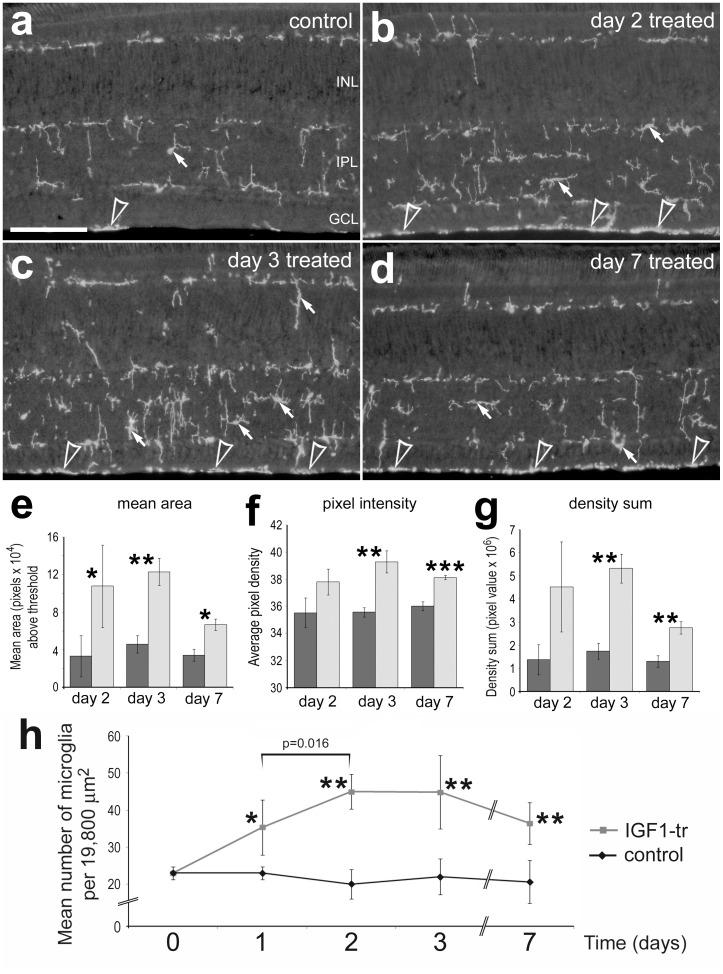
IGF1-treatment causes the sustained reactivity of microglia within the retina. Retinal sections were labeled with antibodies to CD45 (**a**–**d**). Arrows indicate the somata of microglia within the retina and arrow-heads indicate CD45-positive processes and/or cells at the ILM. Images of control and treated retinas were obtained using identical microscope, camera settings, and post-acquisition processing settings. The scale bar (50 µm) in panel **a** applies to **a**–**d**. The histograms in panels **e**–**g** illustrate levels of CD45-immunfluorescence in retinas at 2, 3 and 7 days after IGF1-treatment. ImagePro 6.2 was used to measure total area (**e**) for pixel intensities >72 for CD45 (0 = black, 255 = saturated), mean pixel intensity (**f**) above threshold, and the density sum (**g**; total values of pixels within thresholded objects). Measurements of CD45-immunofluoresence included all layers across 640 µm of linear retina (12 µm-thick section). The plot in panel **h** illustrates the mean (±SD) number of microglial somata (CD45+ DRAQ5 or ToPro3) per 19,800 µm^2^ of retina (n = 5 for each time point). Significance of difference among groups were determined by using a one-way ANOVA (p<0.0001). Significant of different between control and treated groups (*p<0.05, **p<0.001, ***p<0.0001), and between different treatment time-points (brackets and p-values) was determine by using a two-tailed post-hoc Student’s t-test. Abbreviations: ONL – outer nuclear layer, INL – inner nuclear layer, IPL – inner plexiform layer, GCL – ganglion cell layer.

### Effects of IGF1 on the Expression of Nkx2.2, Sox9 and Sox2 in NIRG Cells

Previous reports have demonstrated that IGF1 appears to increase levels of Sox2 and Sox9 in Müller glia [8], and that Nkx2.2 appears elevated in NIRG cells in NMDA-damaged retinas [15]. Accordingly, we sought to measure levels of Nkx2.2, Sox9 and Sox2 in NIRG cells treated with IGF1. We restricted our analysis to NIRG cells that were located in the IPL or proximal INL, because unambiguous identification of NIRG cells in the GCL is complicated by the expression of Sox9 and Nkx2.2 by oligodendrocytes [8]. To measure levels of expression within the NIRG cells separately from those in other types of retinal glia, we used quantitative immunofluoresence for Sox2, Sox9 and Nkx2.2 in the nuclei of NIRG cells found in the IPL, similar to previous reports [8], [16], [17], [18], [19]. Although two consecutive daily injections of 800 ng IGF1 had no significant effects upon levels of Sox2 (Figs. 2a and 2b) and Sox9 (not shown) in the NIRG cells, we found a significant increase in the levels of Nkx2.2 (Figs. 2c–e). Quantitative immunofluorescence revealed significant increases in the mean pixel intensity, area and density sum per Nkx2.2-positive nucleus in the IPL of IGF1-treated retinas (Fig. 2e).

**Figure 5:**
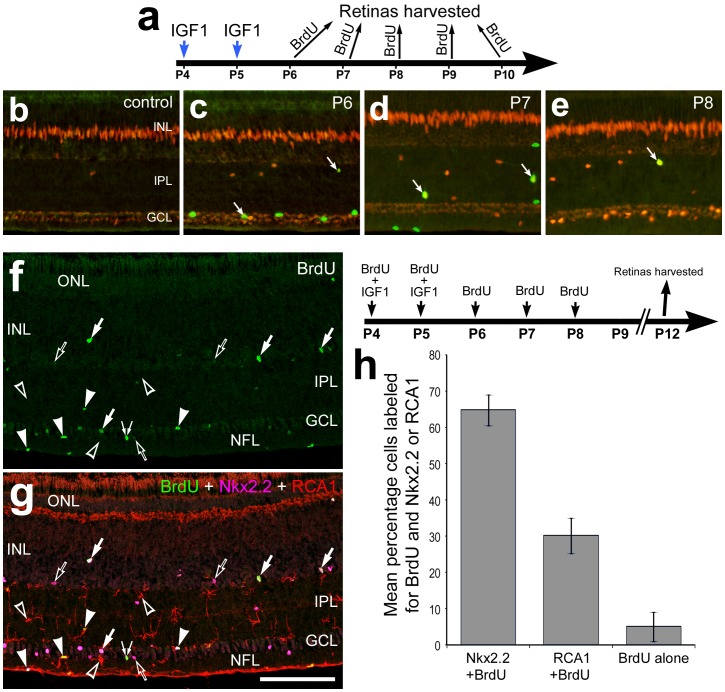
NIRG cells and microglia continue to proliferate for several days after exposure to IGF1. Vertical sections of the retina were labeled for Sox9 (red; **b**–**e**), BrdU (green; **b**–**g**), Nkx2.2 (magenta; **g**), and RCA1 (red; **g**). (**a**) Retinas were obtained from eyes that received injections of IGF1 at P4 and P5, and a single dose of BrdU was applied 4 hrs before harvesting at P6 (**c**), P7 (**d**), P8 (**e**), P9 and P10 (not shown). Alternatively, eyes received injections of IGF1+BrdU at P4 and P5, BrdU alone at P6, P7 and P8, and retinas harvested at P12 (**f**–**h**). Wide-field microscopy (**b**–**e**) and confocal microscopy were used to obtain images (**f** and **g**). The scale bar (50 µm) in panel **g** applies to **b**–**g**. Arrows indicate NIRG cells labeled for BrdU and Sox9 (**b**–**e**) or BrdU and Nkx2.2 (**f** and **g**), hollow arrows indicate Nkx2.2-positive NIRG cells that are BrdU-negative, arrow-heads indicate BrdU-labeled RCA1-positive microglia, hollow arrow-heads indicate BrdU-negative RCA1-positive microglia, and small double arrows indicate BrdU-labeled cells that are negative for RCA1 and Nkx2.2 (**f** and **g**). Abbreviations: ONL – outer nuclear layer, INL – inner nuclear layer, IPL – inner plexiform layer, GCL – ganglion cell layer.

### Transient Effects of IGF1 on NIRG Cells

We have reported previously that intraocular injections of IGF1 stimulates the NIRG cells to proliferation, up-regulate the intermediate filament transitin, and migration of into distal layers of the retina [8]. However, it remains uncertain whether the effects of IGF1 on the numbers and distribution of NIRG cells are short-lived or long-lasting. Accordingly, we tested whether NIRG cells continue to proliferate and accumulate in the days following IGF1-treatment. Consistent with a previous report [8], one day after treatment with IGF1 there was a significant increase in the number of NIRG cells within the IPL; numbers of NIRG cells within the IPL declined over the following days (Figs. 3a–d). At 2 and 3 days after IGF1-treatment, numbers of NIRG cells remained elevated with increased numbers of cells appearing within the IPL (Figs. 3a–d). Within the INL, the NIRG cells were significantly more abundant at 2 days after treatment, and peaked in abundance at 3 days after treatment (Fig. 3d). Interestingly, at 7 days after IGF1-treatment, numbers of NIRG cells within the IPL and INL returned to levels seen in control, untreated retinas (Figs. 3a–f). Immunofluorescence for transitin indicated a transient up-regulation of this intermediate filament in IGF1-treated NIRG cells (Figs. 3e–i). In parallel to the accumulation of the NIRG cells, levels of transitin appeared most elevated at 1 day after treatment, and remained elevated at 2 and 3 days after treatment (Figs. 3e–h). By comparison, levels of transitin in the NIRG cells appeared to decrease to levels seen in control retinas at 7 days after IGF1-treatment (Figs. 3i).

**Figure 6:**
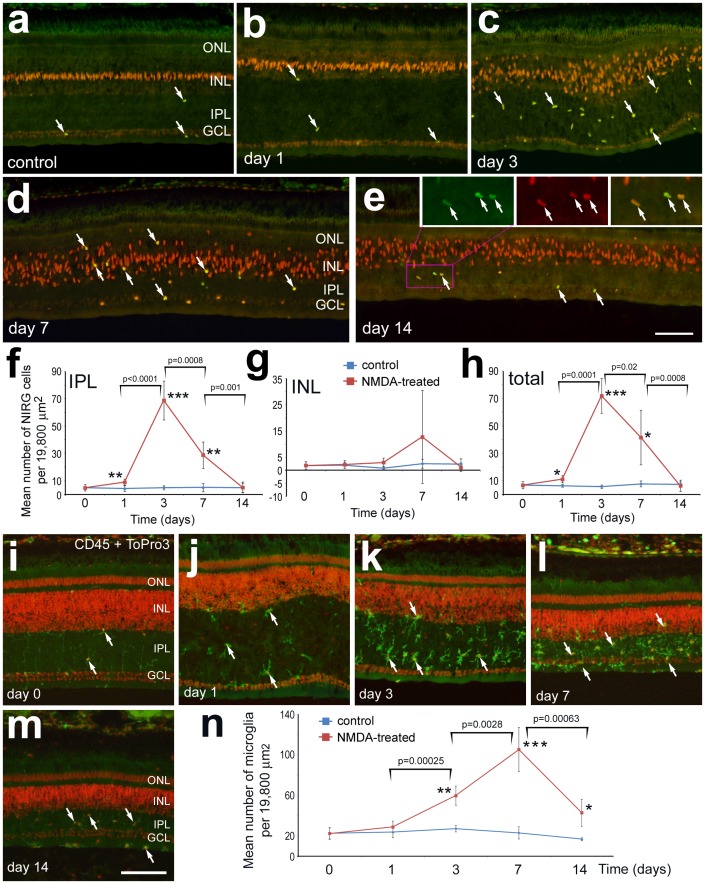
NIRG cells and microglia transiently accumulate in the retina following NMDA-induced excitotoxic damage. Retinas were obtained from eyes treated with NMDA at P7 and harvested at P8 (day 1), P10 (day 3), P14 (day 7) and P21 (day 14). Vertical sections of the retina were labeled with antibodies to Nkx2.2 (green) and Sox9 (red) (**a**–**e**), or CD45 (green) and ToPro3 (red) (**i**–**m**). Arrows indicate the nuclei of NIRG cells that are labeled for Nkx2.2 and Sox9 (**a**–**e**) or CD45-positive microglia (**i**–**m**). Plots illustrate the mean (±SD) number of NIRG cells (**f**–**h**) or microglia (**n**) per 19,800 µm^2^. Significance of difference among groups was determined by using a one-way ANOVA (p<0.0001). Significance of difference between control and treated groups (*p<0.05, **p<0.001, ***p<0.0001), and between treatment time-points (brackets and p-values) was determined by using a post-hoc two-tailed Student’s t-test. The scale bar (50 µm) in panel **e** applies to **a**–**e**, and the bar in **m** applies to **i**–**m**. Abbreviations: ONL – outer nuclear layer, INL – inner nuclear layer, IPL – inner plexiform layer, GCL – ganglion cell layer.

Previous studies have indicated that the differentiation, reactivity and proliferation of glial cells is influenced by secreted factors such as EGF and CNTF [20], [21]. Accordingly, we tested whether intraocular injections of EGF and CNTF influence the reactivity and proliferation of NIRG cells. EGF and CNTF appeared to have no effect on the phenotype, migration or accumulation of NIRG cells in the retina (not shown).

**Figure 7:**
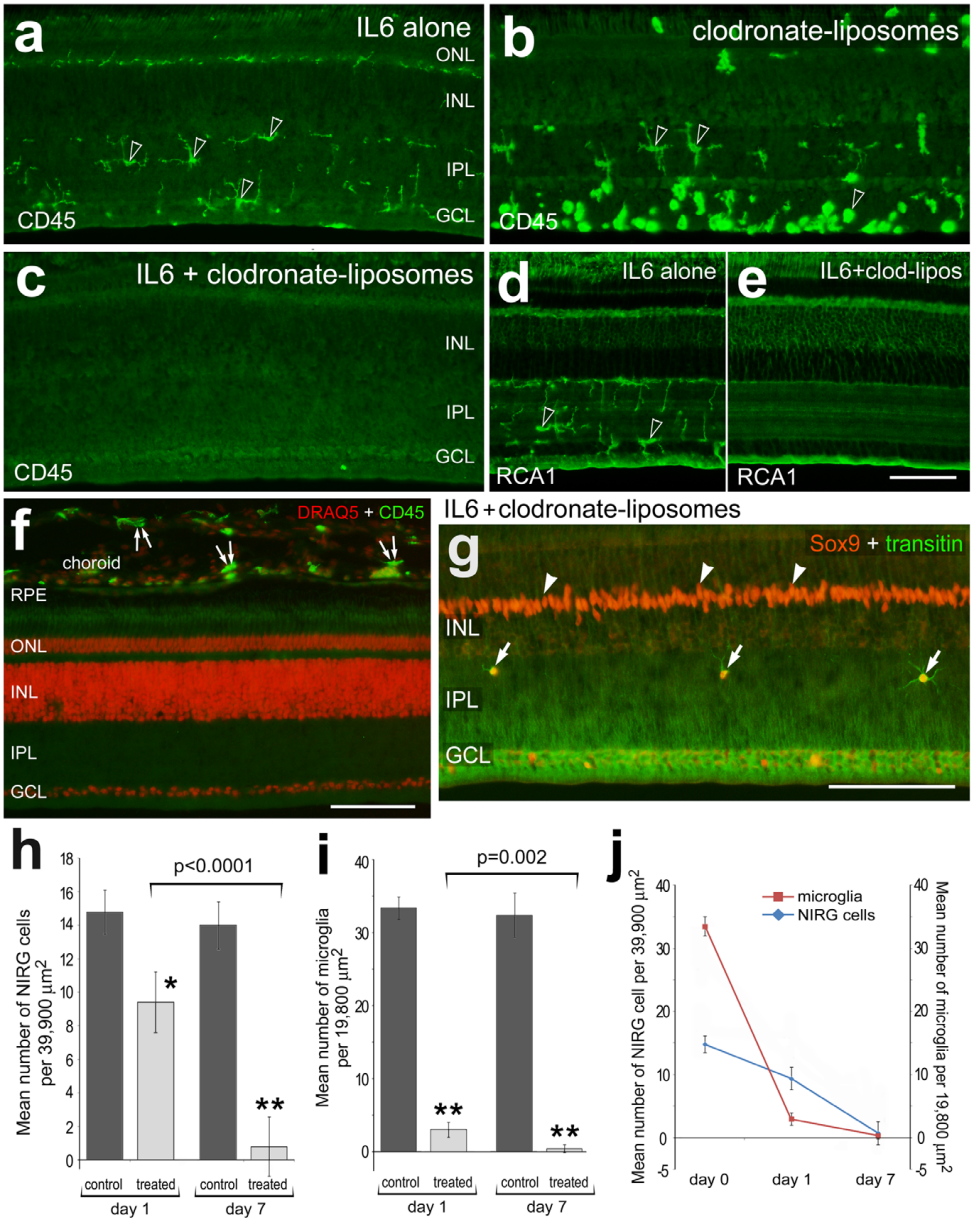
NIRG cells do not survive when the microglia have been selectively ablated by the combination of IL6 and clodronate-liposomes. The dose of clodronate-liposomes was titered-down to the minimum dose required to eliminate >90% of the microglia at 1 days after treatment. Vertical sections of the retina were labeled for CD45 (**a**–**c**), RCA1 (**d** and **e**), CD45 and DRAQ5 (**f**), and transitin and Sox9 (**g**). Retinas were obtained from eyes one day after treatment with IL6 alone (**a** and **d**), clodronate liposomes alone (**b**) or IL6 with clodronate liposomes (**c** and **e**–**g**). Arrows indicate NIRG cells in the IPL, hollow arrow-heads indicate the somata of microglia, arrow-heads indicate the nuclei of Müller glia, and small double-arrows indicate monocytes in the choroid. The scale bar (50 µm) in panel **f** applies to **a**–**c** and **f**, the bar in **e** applies to **d** and **e**, and the bar in **g** applies to **g** alone. The histograms in panels **h** and **i**, and the plot in **j**, illustrate the mean (±SD) number of microglia in the retina (**i** and **j**) or NIRG cells (**h** and **j**) in the IPL and INL at 1 and 7 days after treatment with IL6 alone (control) and IL6 with clodronate-liposomes. Significance of difference was determined by using a one-way ANOVA (p<0.0001). Significance of difference between control and treated groups (*p<0.001, **p<0.0001), and between treatment time-points (brackets and p-values) was determined by using a post-hoc two-tailed Student’s t-test. Abbreviations: RPE – retinal pigmented epithelium, ONL – outer nuclear layer, INL – inner nuclear layer, IPL – inner plexiform layer, GCL – ganglion cell layer.

### Transient Effects of IGF1 on Microglia in Response to IGF1

We have reported previously that at one day after IGF1-treatment, microglia appeared to become reactive, acquiring ameboid morphology and up-regulating expression of CD45 [8]. However, it remains unknown whether the responses of microglia to IGF1 are sustained or transient. The reactivity of microglia was determined by measuring levels of CD45 and assessing the distribution and morphology, similar to previous reports [8], [18]. Similar to the NIRG cells, the microglia were reactive at 2 and 3 days after treatment with IGF1 (Figs. 4a–c and 4e–g). Unlike the NIRG cells, the microglia remained reactive for at least 7 days after IGF1-treatment (Fig. 4a–d). We found significant increases in the area, pixel intensity and density sum of CD45-immunofluoresence at 1, 3 and 7 days after IGF1-treatment (Figs. 4e–g). In addition, there was a noticeable build-up of CD45-immunofluorescence at the inner limiting membrane (ILM) in IGF1-treated retinas (Figs. 4a–d). Numbers of microglia were significantly increased at 1 day after IGF1-treatment, and further increased in abundance at 2 days after treatment (Fig. 4h). The abundance of microglia remained elevated at 3 days after treatment, and numbers were not significantly reduced at one week after treatment (Fig. 4h). The somata of the microglia in IGF1-treated retinas were abundant in the NFL, at the ILM at the vitread surface of the retina (Fig. 4a–d).

**Figure 8:**
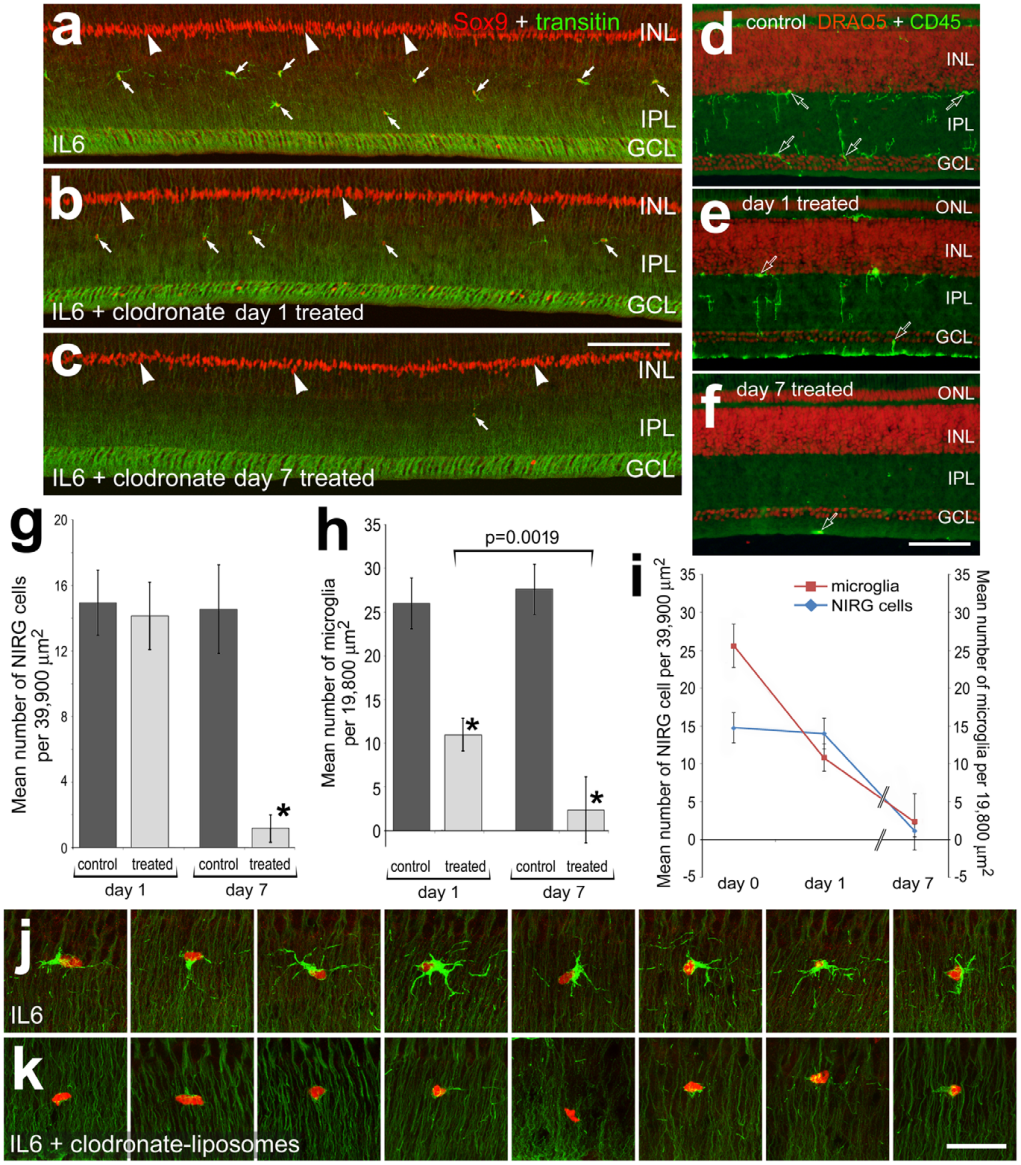
A partial depletion of the microglia influences the phenotype and survival of the NIRG cells. The dose of clodronate-liposomes was titered-down so that nearly one-half of the microglia survived at 1 day after treatment. Retinas were obtained 1 or 7 days after treatment with IL6 alone (control) or IL6 with clodronate-liposomes (treated). Vertical sections of the retina were for Sox9 (red) and transitin (green; **a**–**c**, **j** and **k**), or DRAQ5 (red) and CD45 (green; **d**–**f**). The histogram in **g** illustrates the mean (±SD) illustrates the mean (±SD) number of NIRG cells in the IPL and INL at 1 and 7 days after treatment. The histogram in **h** illustrates the mean (±SD) number of microglia in the retina at 1 and 7 days after treatment. The plot in **i** combines the data from the histograms in **g** and **h** to demonstrate the depletion of microglia and NIRG cells over time following treatment with IL6/clodronate-liposomes. Panels **j** and **k** include representative high-magnification images of representative NIRG cells within the IPL of control (IL6 alone) or treated (IL6+ clodronate-liposomes) retinas at 1 day after treatment. Images were obtained using confocal (**a**–**c**, **j** and **k**) and widefield (**d**–**f**) microscopy. Images of control and treated retinas were obtained using identical microscope, camera settings, and post-acquisition processing settings. Arrows indicate NIRG cells, hollow arrows indicate the somata of microglia, and arrow-heads indicate the nuclei of Müller glia. The scale bar (50 µm) in panel **c** applies to **a**–**c**, and the bar (10 µm) in **k** applies to **j** and **k**. Significance of difference was determined by using a one-way ANOVA (p<0.0001). Significance of difference between control and treated groups (*p<0.0001), and between treatment time-points (bracket and p-values) was determined by using a post-hoc two-tailed Student’s t-test. Abbreviations: ONL – outer nuclear layer, INL – inner nuclear layer, IPL – inner plexiform layer, GCL – ganglion cell layer.

### IGF1-induced Proliferation of Microglia and NIRG Cells

To determine whether the accumulation of NIRG cells and microglia in IGF1-treated retinas involves on-going cell proliferation we probed for BrdU-labeled cells. With BrdU applied 4 hrs prior to harvest, we identified proliferating NIRG cells at 1, 2 and 3 days after IGF1 treatment (Figs. 5b–e). By comparison, we failed to find BrdU-labeled NIRG cells at 4 or 5 days after IGF1-treatment (data not shown). When BrdU was applied with the IGF1 and at 1, 2 and 3 days after treatment, we found that the majority (about 65%) of BrdU-labeled cells were NIRG cells (Fig. 5h). By comparison about 30% of the BrdU-labeled cells were microglia (Fig. 5h). The identity of the remaining 5% of BrdU-labeled cells remains unknown. These findings are consistent with the hypothesis that the proliferation underlies the accumulation of NIRG cells and microglia in IGF1-treated retinas.

**Figure 9:**
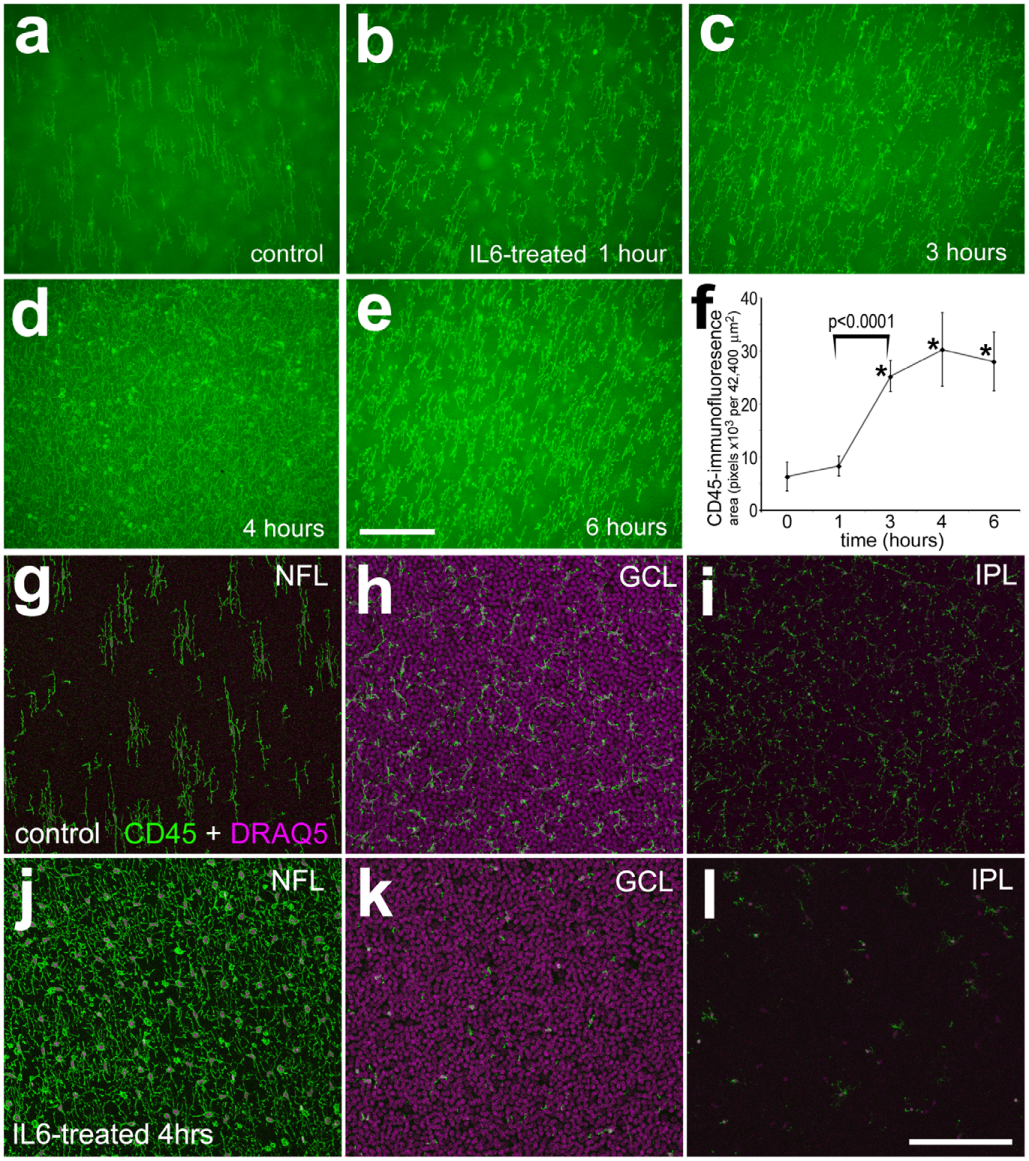
IL6 stimulates the rapid migration of microglia to vitread surface of the retinas. Retinas were obtained from eyes injected with vehicle (control; **a**, **g**–**i**) or IL6 at 1 (**b**), 3 (**c**), 4 (**d**, **j**–**l**) and 6 (**e**) hours after treatment. Whole-mount preparations of the retina were labeled with DRAQ5 (magenta; **g**–**i**) and antibodies to CD45 (**a**–**e** and **g**–**l**). The scale bar (50 µm) in panel **e** applies to **a**–**e**, and the bar in **l** applies to **g**–**l**. The histogram in panel **f** illustrates the mean (±SD) area for CD45-immunofluorescence above threshold, per field of view (42,400 µm^2^), at 1, 3, 4 and 6 hours after treatment. Image Pro 6.2 was used to measure the total area for pixel intensities >68 (0 = black, 255 = saturated green) per field of view, as described in the Methods. Significance of difference was determined by using a one-way ANOVA (p<0.0001). Significance of difference between control and treated groups (*p<0.0001), and between treatment time-points (bracket and p-values) was determined by using a post-hoc two-tailed Student’s t-test. Abbreviations: ONL – outer nuclear layer, INL – inner nuclear layer, IPL – inner plexiform layer, GCL – ganglion cell layer, NFL – nerve fiber layer.

### Transient Accumulation of NIRG Cells in Damaged Retinas

Prior to a definitive identification of NIRG cells, we observed a significant accumulation of Nkx2.2-labeled cells in the IPL of NMDA-damaged retinas [15]. Given that there is a transient accumulation of NIRG cells in IGF1-treated retinas (see Fig. 2), we sought to determine whether the accumulation of NIRG cells in damaged retinas was transient or permanent. We found small, but significant, accumulations of NIRG cells in the IPL of NMDA-damaged retinas at 1 day after treatment (Figs. 6a, b, and f–h). At 3 days after treatment, we found a large increase, more than 7-fold, in the number of NIRG cells in the IPL of damaged retinas (Figs. 6c and 6f). At 7 days after treatment, the numbers of NIRG cells in the retina, namely the IPL, remained significantly elevated compared to control retinas, but the abundance of the NIRG cells was reduced compared to 3 days after treatment (Figs. 6d, f and h). At 7 days after treatment, numbers of NIRG cells were not significantly more abundant in the INL, although there was large variability between individuals and NIRG cells were often observed deep within the INL near the OPL (Figs. 6d and g). By 14 days after treatment, numbers of NIRG cells were significantly reduced compared to 7 days after treatment, and levels were not significantly different from those seen in control retinas (Fig. 6e, f–h). Although levels of Sox9 and Nkx2.2 appeared to differ between cells, all NIRG cells expressed detectable levels of both Sox9 and Nkx2.2 (Fig. 6e).

**Figure 10:**
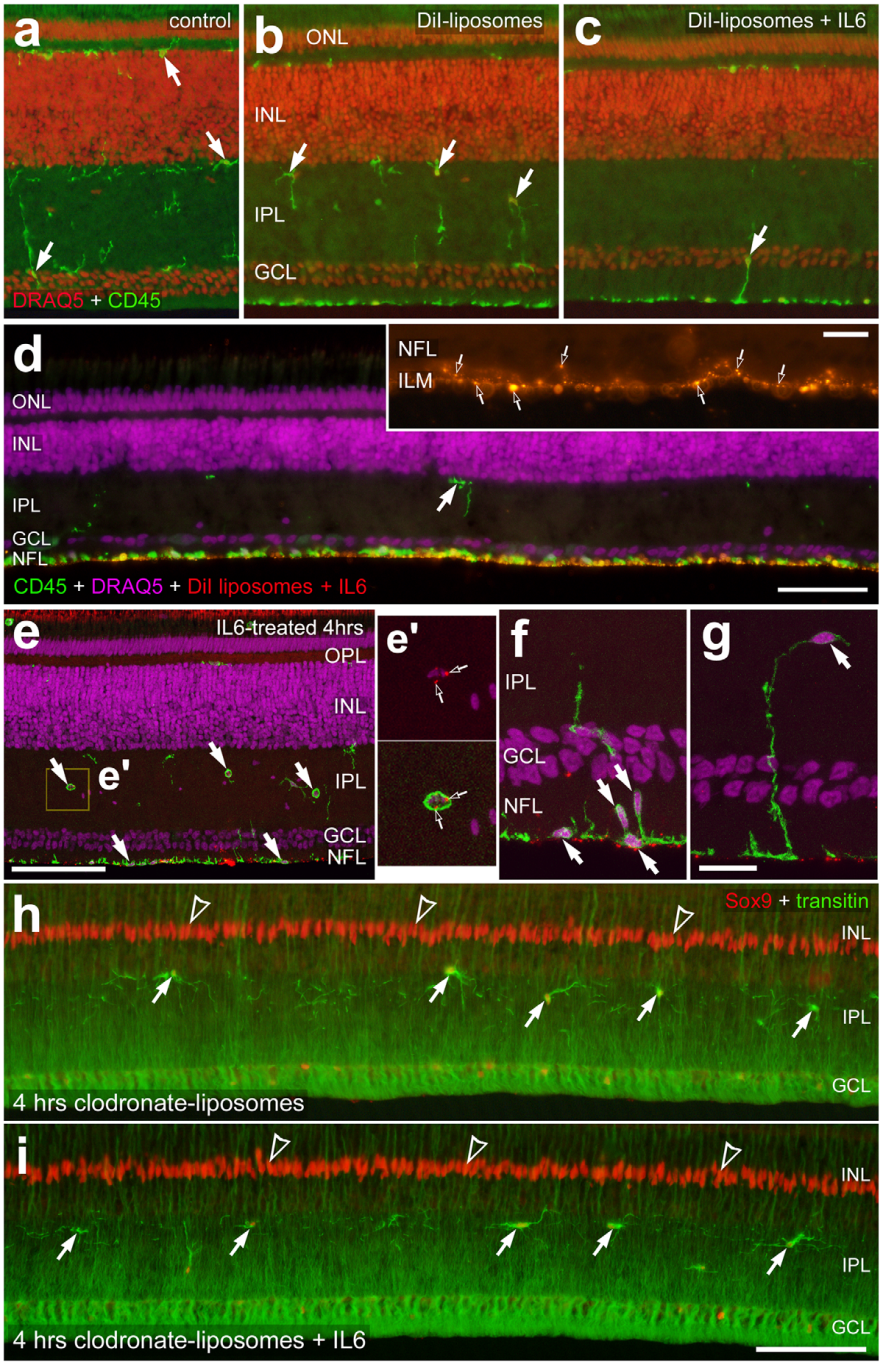
Clodronate-DiI-liposomes accumulate, along with the microglia, at the vitread surface of the retina, whereas the NIRG cells appear unaffected by short-term exposure to IL6 and liposomes. Vertical sections of the retina were labeled with DRAQ5 (red, **a**–**c**; magenta, **d**–**g**) and antibodies to CD45 (green, **a**–**g**), Sox9 (red, **h** and **i**), and transitin (green, **h** and **i**). The red fluorescent puncta in panels **d**–**g** are DiI-labeled liposomes. Images were obtained by using wide-field (**a**–**c**, inset in **d**, **h** and **i**) or confocal (**d**–**g**) microscopy. Images were obtained from eyes that were treated with saline (**a**), clodronate-DiI-liposomes (**b** and **h**), or the combination of clodronate-DiI-liposomes and IL6 (**c**–**g** and **i**). Arrows indicate the somata of microglia or NIRG cells, hollow arrows indicate DiI-liposomes, and hollow arrow-heads indicate the nuclei of Müller glia. The scale bar (50 µm) in panel **d** applies to **d** alone, the bar in **e** applies to **a**–**c** and **e**, the bar in **i** applies to **h** and **I**, and the bar (10 µm) in the inset in panel **d** applies to the inset alone. Abbreviations: ONL – outer nuclear layer, OPL- outer plexiform layer, INL – inner nuclear layer, IPL – inner plexiform layer, GCL – ganglion cell layer, NFL – nerve fiber layer, ILM – inner limiting membrane.

The time-course of accumulation and reactivity of microglia in NMDA-damaged retinas was similar that of NIRG cells. At 1 day after NMDA-induced damage, the microglia appeared reactive, but there was no significant increase in the number of microglia (Figs. 6i, j and n). In damaged retinas, microglia were absent from the OPL (8.0±1.9 vs 0.0±0.0 microglia in the OPL per 19,800 µm^2^; n = 5), suggesting a vitread migration. At 3 days after treatment, numbers of microglia were significantly increased by nearly 3-fold (Figs. 6k and n). At 7 days after treatment, numbers of microglia were more than 5-fold that of control levels (Figs. 6l and n). The microglia were concentrated within the IPL at 3 and 7 days after treatment (Figs. 6k and l). At 14 days after treatment, numbers of microglia were significantly reduced compared to numbers seen at 7 days after treatment, but numbers of microglia remained significantly elevated compared to controls (Figs. 6m and n).

**Figure 11:**
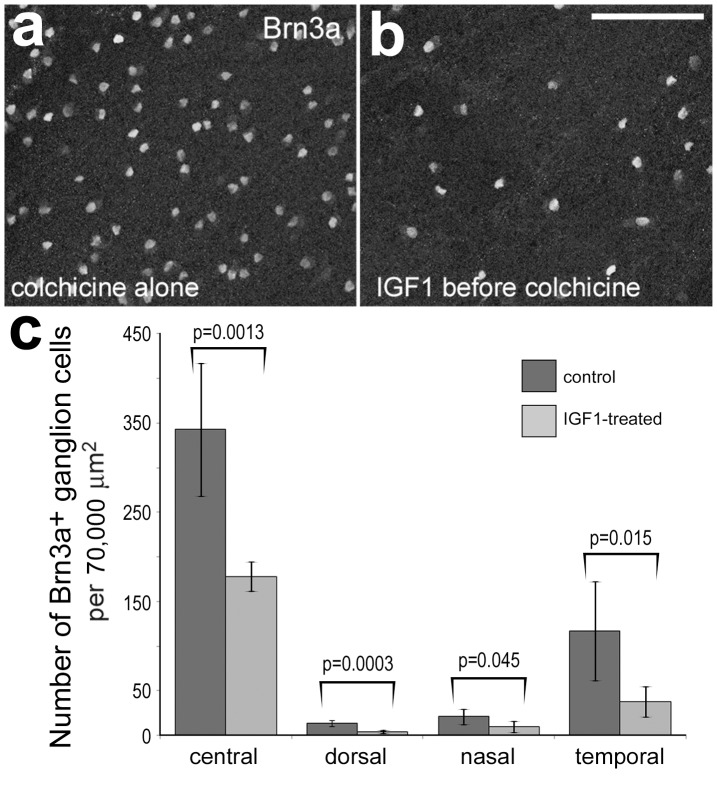
IGF1-treatment reduced the survival of colchicine-damaged ganglion cells. Retinas were obtained from eyes that were injected with saline (**a**) and colchicine (**b**) 10 days after treatment. Whole-mount preparations of the retina were labeled with antibodies to Brn3a to identify the nuclei of ganglion cells. Panel **c** is a histogram illustrating the mean number of Brn3a-positive ganglion cells per 70,000 µm^2^ in central and peripheral regions of the retina. Significance (p-values) of difference between numbers of Brn3a-positive ganglion cells in control and treated retinas was determined by using a two-tailed Student’s t-test. The scale bar (50 µm) in panel **b** applies to panels **a** and **b**.

### The Survival of NIRG Cells Depends on Microglia

Our findings suggest that the accumulation and reactivity of NIRG cells in response to IGF1 and NMDA-induced damage is paralleled by the accumulation and reactivity of the microglia. Thus, we sought to examine what happens to the NIRG cells when the microglia are selectively ablated. We used clodronate-filled liposomes to selectively ablate the microglia, similar to previous descriptions [22], [23], [24]. We found that application of clodronate liposomes alone did not ablate the microglia, but instead stimulated microglial reactivity (Figs. 7a and 7b). Microglia survived and remained reactive for at least 7 days after treatment with clodronate liposomes alone (not shown). By comparison, application of a single dose of IL6 with the clodronate liposome resulted in the near-complete ablation of microglia, labeled for CD45 or RCA1, within 24 hours of treatment (Figs. 7a and 7c–e). In most instances (9/10), we observed a complete ablation of microglia, whereas CD45-positive monocytes in the choroid appeared unaffected (Fig. 7f). On average, more than 90% of the microglia were ablated at one day after treatment, and nearly 100% of the microglia were ablated at 7 days after treatment (Figs. 7a,c,h and j). Treatment with IL6/clodronate-liposomes had no detectable effects upon Müller glia; we failed to detect changes in Müller glial expression of GFAP, transitin, pERK1/2, p38 MAPK, Egr1, cFos or pCREB (data not shown). Surprisingly, significant numbers of NIRG cells were lost from retinas treated with IL6/clodrondate-liposomes. At one day after treatment with IL6/clodronate-liposomes, numbers of NIRG cells were reduced by approximately one-third (Figs. 7g, I and j). At 7 days after treatment, less than 5% of the NIRG cells remained within the retina (Figs. 7h and 7j).

**Figure 12:**
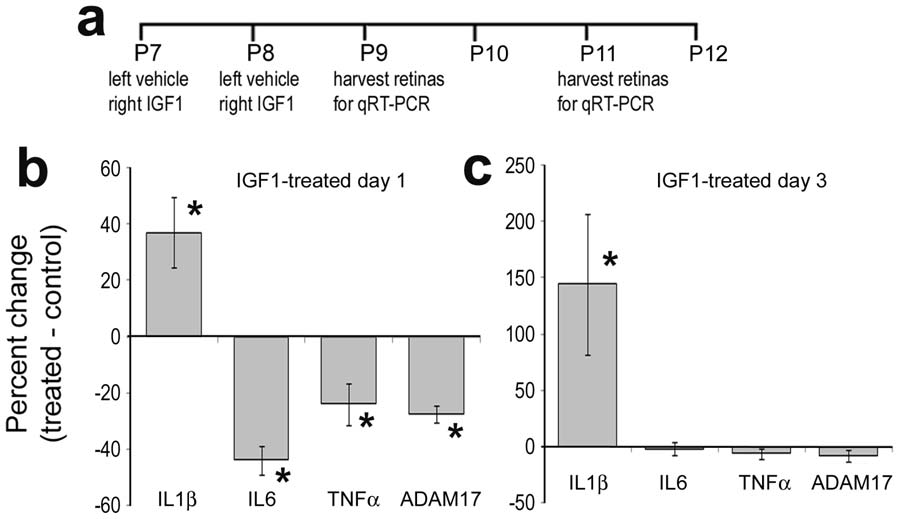
Levels of pro-inflammatory cytokines fluctuate in retinas treated with IGF1. qRT-PCR was used to measure levels of mRNA in retinas treated with IGF1 at 1 and 3 days after treatment. qRT-PCR was used to measure retinal levels of IL1β, IL6, TNFα and ADAM17. Significance (*p<0.01) of difference between mRNA levels in control and IGF1-treated retinas was determined by using a two-tailed Student’s t-test.

To further study the relationship between the microglia and NIRG cells, we titered-down doses of clodronate-liposomes to spare about one-half of the microglia at one day after treatment, and probed for the phenotype and numbers of NIRG cells. At one day after treatment with IL6 and clodronate-liposomes, there was no significant depletion in the number of NIRG cells within the IPL, despite a loss of more than half of the microglia (Figs. 8a–i). However, in this same cohort of animals, when examined at 7 days after treatment, there was a massive depletion of NIRG cells, which paralleled the loss of microglia; nearly 95% of the NIRG cells and microglia were lost (Figs. 8a–i).

The phenotype of the NIRG cells was affected by the loss of microglia resulting from treatment with IL6/clodronate-liposomes (Figs. 8j and k). At one day after treatment with IL6/clodronate-liposomes, the NIRG cells remaining in the IPL appeared to have decreased levels of transitin and fewer peripheral processes (Figs. 8j and k). IL6 alone had no effects upon the phenotype of NIRG cells (data not shown).

### IL6 Stimulates the Rapid Migration of Microglia to the Vitread Surface of the Retina Where Clodronate-liposomes Accumulate

To better understand how the IL6/clodronate-liposomes influence the microglia and NIRG cells, we labeled the liposomes with a fluorescent dye (DiI) and made short-term observations in retinal whole-mounts and sections. In control retinas, microglia were tiled across the vitread surface of the retina, and scattered across the GCL and IPL (Figs. 9a, g–i). A single intravitreal injection of IL6 caused a rapid accumulation of microglia to the vitread surface of the retina. The CD45-immunofluorescence at the vitread surface of the retina was significantly increased between 1 and 3 hours after treatment, and remained elevated for at least 6 hours (Figs. 9a–f). Confocal analysis indicated an accumulation of CD45-positive microglia at the vitread surface, and a depletion of microglia from the GCL and IPL (Figs. 9g–l).

Short-term exposure (4 hrs) to clodronate-liposomes alone stimulated the reactivity of microglia and caused the accumulation of microglia at the vitread surface of the retina (Figs. 10a and 10b). By comparison, short-term exposure to clodronate liposomes *and* IL6 enhanced the reactivity and vitread accumulation of microglia compared to treatment with liposomes alone (Figs. 10a–c). The vast majority of DiI-labeled liposomes were detected at the vitread surface, at the inner limiting membrane of the retina, whereas relatively few DiI-liposomes were found within in the NFL (Figs. 10d–g). The microglia at the vitread surface of the retina were often observed in close proximity to DiI-liposomes (Figs. 10d–g). The DiI-liposomes were rarely observed within the GCL or IPL (Figs. 10d–g). Within the GCL and IPL, the DiI-label was always associated with CD45-positive microglia (Fig. 10d). At 4 and 6 hours (not shown) after treatment with clodronate liposomes, with or without IL6, the NIRG cells appeared unaffected (Figs. 10h and 10i and not show). The reactivity, number and distribution of NIRG cells were unaffected by treatment with liposomes alone or the combination of liposomes and IL6 (Fig. 10h and 10i).

### IGF1-treatment Diminished the Survival of Ganglion Cells in Damaged Retinas

We have reported previously that insulin- or IGF1-mediated activation of NIRG cells and microglia increases the susceptibility of amacrine and bipolar neurons to excitotoxic damage [8], [18]. It remains uncertain whether treatment of the retina with IGF1 impacts the survival of retinal neurons in addition to amacrine and bipolar neurons. Accordingly, we examined whether IGF1-treatment influences the survival of ganglion cells in retinas damaged by colchicine. Colchicine is known to damage and destroy ganglion cells during early postnatal development of the chick retina [25], [26]. IGF1-treatment significantly diminished, by more than 50%, the survival of colchicine-damaged Brn3a-positive ganglion cells in central, dorsal, nasal and temporal regions of the retina (Fig. 11).

### IGF1 Influences Retinal Levels of Cytokines

Given that IGF1 influences the reactivity of retinal microglia and diminishes the survival of neurons in damaged retinas, we postulated that IGF1 may influence retinal levels of pro-inflammatory cytokines. Accordingly, we used qRT-PCR to measure retinal levels of mRNAs for interleukin 1β (IL1β), IL6, tumor necrosis factor-α (TNFα) and the TNFα–processing enzyme ADAM17. One day after the last injection of IGF1, we found that levels of IL1β were significantly elevated, whereas levels of IL6, TNFα and ADAM17 were significantly reduced (Figs. 12a and 12b). At 3 days after the last injection of IGF1, levels of IL1β were further elevated, whereas levels of IL6, TNFα and ADAM17 were not significantly different from controls (Figs. 12a and 12c). It must be noted that the qRT-PCR measures of IL1β mRNA may not directly indicate levels of the mature active cytokine.

## Discussion

We report here that the effects of IGF1 on the NIRG cells are transient. The transient IGF1-mediated responses of NIRG cells include proliferation, accumulation, distal migration, and up-regulation of transitin. Similar to the effects of IGF1, NMDA-induced retinal damage causes the transient accumulation, distal migration and reactivity of the NIRG cells. These findings suggest that there are homeostatic mechanisms in place within the retina to maintain the numbers, distribution and phenotype of the NIRG cells. Further, we find that the responses of the NIRG cells to IGF1 and damage are paralleled by those of microglia. We find that the survival of NIRG cells may depend upon the microglia; the NIRG cells perish in retinas where the microglia are selectively ablated. We have reported previously, using *in situ* hybridization, that IGF1-receptors are expressed by cells scattered across the IPL [8]. Thus, it seems likely that both NIRG cells and microglia express IGF1-receptors and that IGF1 acts directly on these cells. By contrast, the effects of NMDA on the microglia and NIRG cells are likely to be indirect; studies have failed to provide evidence that NMDA receptors are expressed by cells scattered in the IPL [27], [28], [29].

Collectively, our findings suggest that NIRG cells that are stimulated by IGF1 continue to accumulate, proliferate and migrate distally into the retina for several days after exposure to IGF1. IGF1 is known to have a short half-life (<10 minutes) in serum (reviewed by Torrado and Carrascosa [30]). Although there is currently no data regarding the half-life of IGF1 in the eye, it is expected to be relatively short (<30 minutes), similar to that of other growth factors such as Fibroblast Growth Factors [31]. Thus, we predict that short-term exposure to IGF1 could have long-lasting effects that persist for several days, upon the reactivity and phenotype of the NIRG cells. Some of the long-lasting effects of IGF1 may occur through a secondary increase in the pro-inflammatory cytokine IL1β. Our findings indicate that retinal levels of IL1β are elevated for at least 3 days after IGF1-treatment. It remains uncertain whether IL1β influences the proliferation, migration and reactivity of NIRG cells.

Numbers of NIRG cells may be regulated by feed-back, homeostatic mechanisms that are intrinsic to the retina. Our data suggest that the increased numbers of NIRG cells that are produced in response to IGF1 are pruned back to control levels by 7 days after treatment. However, we failed to detect dying cells, labeled for TUNEL or cleaved caspase 3, in IGF1-treated retinas between 4 and 7 days after treatment (unpublished observations). It is possible that the relatively low abundance of these cells and the narrow window of time that dying cells can be identified make it unlikely to detect dying NIRG cells. Nevertheless, total numbers of NIRG cells within the IPL return to normal 7 days after IGF1-treatment. These findings suggest that there are retina-intrinsic mechanisms that regulate numbers of NIRG cells within the IPL. It is known that IGF1 influences the developmental accumulation of glial cell in the CNS [32]. It is also possible that Notch- and Jak/Stat-signaling regulate glial numbers beyond development [33]. For example, Notch-signaling is maintained in mature Müller glia, and this pathway influences the proliferation, formation of glia-derived retinal progenitors, and differentiation of glia-derived cells [17], [34]. It remains uncertain whether Notch-signaling influences numbers of NIRG cells in the retina.

Our data suggest that the survival and accumulation of NIRG cells within the retina is linked to the microglia. During embryonic development, microglia begin to appear within the central regions of the quail retina between E8 and E9 [35], [36], equivalent to E9 to E10 in chick development. By comparison, the NIRG cells (or NIRG cell precursors) begin to migrate into the chick retina at about E12 [11]. Thus, the NIRG cells accumulate and ramify within the retina shortly after the microglia, consistent with the hypothesis that the persistence of NIRG cells relies upon the microglia. Further evidence to support this hypothesis comes from findings that the transient accumulation of NIRG cells follows that of microglia in retinas treated with IGF1 or damaged by NMDA. In addition, we find that the selective ablation of the microglia results in the subsequent loss of NIRG cells. Collectively, these findings suggest that the homeostatic mechanisms that regulate the reactivity and numbers of NIRG cells are linked to the microglia within the retina.

Numerous studies have shown that the activities of different types of glial cells in the CNS are coordinated. In the brain there is significant evidence that the activity of astrocytes is intimately associated with the activity of microglia (reviewed by Farina and colleagues [37]). Similarly, in damaged retinas microglia and Müller glia are known to be activated in a coordinated manner. For example, in a rodent model of retinal detachment, Müller glia up-regulate the expression of monocyte chemoattractant protein 1 (MCP1) to facilitate the accumulation of microglia in the distal retina [38]. Similarly, retinal damage is known to stimulate Müller glia to produce TNFα [39]; TNFα is known to stimulate glial reactivity and can also be produced by activated microglia and astrocytes [40]. Although the NIRG cells and microglia appear to be activated in a coordinated manner, we failed to find elevated levels of TNFα in IGF1-treated retinas. The coordinated reactivity and proliferation of NIRG cells and microglia may, in part, be regulated by cytokines other than TNFα, such as IL1β. Our data suggest that the survival of NIRG cells within the retina is somehow dependant on the microglia; this dependence may involve an exchange of cytokines. To the best of our knowledge, there are no reports demonstrating the dependence of the neuroephithelium-derived glial cells upon microglia.

It remains uncertain why the NIRG cells perish following the ablation of microglia in retinas treated with IL6/clodronate-liposomes. It is possible that the NIRG cells phagocytize relatively small amounts of the clodronate-loaded liposomes or phagocytize the remnants of dying microglia that are laden with clodronate. However, there is little evidence that neuroepithelium-derived glia are capable of phagocytosis [41], although there are some reports that Müller glia can be phagocytic [42], [43]. In addition, the NIRG cells never express lysosomal membrane glycoprotein, which is present at low levels in normal microglia and becomes highly expressed by activated microglia [8]. These observations suggest that the NIRG cells do not form primary lysosomes to enable phagocytosis. We found, using a single intraocular injection of DiI-labeled clodronate-liposomes, that microglia, but never NIRG cells, become closely associated with liposomes at the vitread surface of the retina shortly after treatment. Our findings suggest that the microglia rapidly migrate to the vitread surface of the retina to phagocytize clodronate-liposomes; the liposomes do not penetrate with abundance beyond the NFL. The rare DiI-liposomes, or remnants of liposomes, that are detected within IPL are always associated with microglia, suggesting that the microglia migrate back into the retina after being drawn to the vitread surface to phagocytize the liposomes. Unlike the microglia, the NIRG cells appear unaffected shortly after treatment with IL6/clodronate-liposomes. These findings support the notion that the loss of NIRG cells is secondary to the loss of microglia resulting from treatment with IL6/clodronate-liposomes. Additional studies are required to unambiguously determine whether the survival of NIRG cells requires trophic signals derived from microglia.

The stimulation of NIRG cells and microglia by IGF1 decreases the survival of ganglion cells in response to colchicine-mediated damage. It is possible that elevated retinal levels of the pro-inflammatory cytokine IL1β, which occur secondary to IGF1-treatment, influence microglial reactivity and attenuated survival of ganglion cells in damaged retinas. This is consistent with previous findings that IGF1-mediated stimulation of microglia and NIRG cells renders Müller glia, amacrine and bipolar cells more susceptible to an excitotoxic insult [8]. It is believed that colchicine-mediated disassembly of microtubules prevents the retrograde transport of trophic signals in projection neurons, and thereby causes the death of retinal ganglion cells [26]. The mechanisms underlying the diminished survival of ganglion cells in IGF1-treated, colchicine-damaged retinas remain uncertain. We find that IGF1 transiently down-regulates pro-inflammatory cytokines, with the exception of IL1β which remains elevated at 1 and 3 days after treatment. It seems likely that elevated levels of IL1β would stimulate the reactivity of microglia. IL1β is known to stimulate the reactivity of microglia in different regions of the brain and retina [44], [45], [46]. Further, IL1β is known to influence the survival of retinal neurons, including ganglion cells [47], [48], [49]. Thus, it is possible that elevated IL1β impacts the survival of ganglion cells in retinas treated with IGF1 and colchicine.

### Conclusions

We conclude that there are homeostatic mechanisms in place to maintain the phenotype, number and distribution of NIRG cells within the retina. Our data indicate that the reactivity, proliferation and distribution of NIRG cells parallels that of microglia in retinas treated IGF1 or acute damage. We conclude that the NIRG cells do not survive within the retina without the microglia. We propose that the survival and abundance of NIRG cells in the retina is linked to the number and activity of microglia. The reactivity of the NIRG cells and microglia may be linked by IL1β. Further studies are required to determine how IL1β-signaling coordinates the activities of NIRG cells and microglia.

## Materials and Methods

### Animals

The use of animals in these experiments was in accordance with the guidelines established by the National Institutes of Health and the Ohio State University. This study was approved by the Ohio State University Institutional Animal Care and Use Committee (IACUC). Newly hatched leghorn chickens (*Gallus gallus domesticus*) were obtained from the Department of Animal Sciences at the Ohio State University and kept on a cycle of 12 hours light, 12 hours dark (lights on at 7∶00 am). Chicks were housed in a stainless steel brooder at about 25°C and received water and Purina^tm^ chick starter ad libitum.

### Preparation of Clodronate-liposomes

The preparation of clodronate liposomes was based on previous descriptions [22], [23], [24]. In short, 500 µg cholesterol and 8 mg egg lecithin were dissolved in chloroform in a round-bottom flask. Some preparations included a few crystals (<1 mg) of DiI (D282; Invitrogen) to label the liposomes. The solution was evaporated with gentle rotation, leaving a white liposome residue. 157 mg dichloromethylene diphosphonate (clodronate) in water was added to the flask and rotated for 10 min. The preparation was sealed under N_2_ and kept at room temperature for 2 hr. Clodronate encapsulation was facilitated by sonication for 3 min and incubation at 4°C overnight. The liposomes were centrifuged at 10,000×g for 15 min and re-suspended in 200 µl PBS. Between 0.5 and 10 µl of clodronate liposomes in PBS were mixed with 100 ng IL6 for injections. Precise quantitation of the clodronate was difficult, because of the stochastic nature of combination of the clodronate and liposomes. Accordingly, we titered doses to levels where 33 to 100% of the microglia were ablated at 1 day after treatment.

### Intraocular Injections

Chickens were anesthetized and eyes were injected as described in previous reports [26], [50]. For all experiments, the left eyes of chicks were injected with the “test” compound and the contra-lateral eyes were injected with vehicle as a control. Compounds were injected in 20 µl sterile saline with 0.05 mg/ml bovine serum albumin added as carrier. Compounds included EGF (100 ng or 15.6 pmol; R&D Systems), CNTF (100 ng or 0.44 pmol; R&D Systems), IGF1 (200–800 ng or 26.1 to 104.6 pmol per dose; R&D Systems), and interleukin 6 (IL6; 100 ng or 3.85 pmol; R&D Systems). Estimated initial maximum concentrations of factors in the vitreous included 100 ng EGF = 26.3 nM, 100 ng CNTF = 0.74 nM, 200 ng IGF1 = 88 nM, 800 ng IGF1 = 352 nM, 100 ng IL6 = 5.2 nM. Two µg of BrdU was included with each injection (treatment and control) to label proliferating cells. Control eyes were injected with vehicle (saline added with 0.05 ml/ml bovine serum albumin and BrdU).

Damage paradigms: Similar to prior studies [8], [19], [50], we used a single injection of 284 µg of N-Methyl-D-aspartate (NMDA) at P7 to elicit excitoxic damage. Retinas were harvested at different times after NMDA-treatment and retinas processed for immunocytochemistry. Similar to prior studies [25], [26], [51], [52], we used a single injection of 250 ng of colchicine at P2 to damage many ganglion cells. Retinas were harvested at P12, 10 days after treatment, when the damaged effects of colchicine are known to have subsided [51], [52].

### Fixation, Sectioning and Immunocytochemistry

Tissues were fixed, sectioned and immunolabeled as described previously [53], [54]. Retinal whole-mount preparations were processed as described previously [51], [55], [56]. Working dilutions and sources of antibodies used in this study included; (1) goat anti-Sox2 was used at 1∶1000 (Y-17; Santa Cruz Immunochemicals); (2) rabbit anti-Sox9 was used at 1∶2000 (AB5535; Chemicon); (3) rabbit anti-Olig2 was used at 1∶200 (AF2418; R&D Systems); (4) mouse anti-CD45 (HIS-C7; Cedi Diagnostic); (5) mouse anti-Brn3a (Pouf4a) was used at 1∶200 (mab1585; Chemicon); (6) mouse anti-Nkx2.2 was used at 1∶50 (74.5A5; Developmental Studies Hybridoma Bank - DSHB); (7) mouse (IgG) anti-transitin was used at 1∶50 (EAP3; DSHB), (8) mouse anti-BrdU was used at 1∶80 (G3B4; DSHB), and (9) rat anti-BrdU was used at 1∶200 (OBT00030S; Serrotec).

None of the observed labeling was due to non-specific binding of secondary antibody or auto-fluorescence because sections labeled with secondary antibodies alone were devoid of fluorescence. Secondary antibodies included donkey-anti-goat-Alexa488/568, goat-anti-rabbit-Alexa488/568/647, goat-anti-mouse-Alexa488/568/647, goat-anti-rat-Alexa488 and goat-anti-mouse-IgM-Alexa568 (Invitrogen) diluted to 1∶1000 in PBS plus 0.2% Triton X-100.

### Labeling for RCA1

Sections of the retina were washed 15 min in PBS +0.1% Tween20. Following 2 additional washes in PBS, sections were incubated under 1.7 µg/ml Biotinylated *Ricinus Communis* Agglutinin I (RCA1; Vector Laboratories) diluted in PBS for 1 h at 38°C in a humidified chamber. Sections were washed in PBS at 38°C, washed in PBS at room temperature, and incubated for 30 min under Streptavidin-Alexa488 (Invitrogen) diluted to 1∶2000. Finally, slides were washed in PBS and then either mounted with coverglass or processed for indirect immunofluorescence.

### Reverse Transcriptase PCR and Quantitative RT-PCR

Retinas from two P7 chicks were pooled and placed in 1.5 ml of Trizol Reagent (Invitrogen; Carlsbad, CA) and total RNA was isolated according to the Trizol protocol and resuspended in 50 µl RNAse free water. Genomic DNA was removed by using the *DNA FREE* kit provided by Ambion (Austin, TX). cDNA was synthesized from mRNA by using Superscript^tm^ III First Strand Synthesis System (Invitrogen) and oligo dT primers according to the manufacturer’s protocol. Control reactions were performed using all components with the exception of the reverse transcriptase to exclude the possibility that primers were amplifying genomic DNA.

PCR primers were designed by using the Primer-BLAST primer design tool at NCBI (http://www.ncbi.nlm.nih.gov/tools/primer-blast/). Primer sequences are as follows: IGFBP1 - forward 5′ TGGCTCGGGCTAGCTGGATG 3′ – reverse 5′ ACCAGCACCCAGCGGAATCT 3′, IGFBP2 - forward 5′ GGTTTGCGCTGCTACCCCGA 3′ – reverse 5′ CCAACGGACAGGGGACAGGC 3′, IGFBP3 - forward 5′ TCCGCCGGTTGCCATGGTGT 3′ – reverse 5′ CTTGCCATCATATCCAGGAAGCGGT 3′, IGFBP4 forward 5′ TGGTGCGTGGACCGCAAGAC 3′ – reverse 5′ AGCGATGGGGGCGTCCCATA 3′, IGFBP5 - forward 5′ TGTGCCTCTGGCAGGGGGTA 3′ – reverse 5′ CAACACAGCCCACGCTTCCG 3′, and GAPDH - forward 5′ GGAACACTATAAAGGCGAGAT 3′ – reverse 5′ TCACAAGTTTCCCGTTCTCA 3′. Predicted product sizes, in base pairs, were 807 (IGFBP1), 1108 (IGFBP2), 820 (IGFBP3), 1017 (IGFBP4), 608 (IGFBP5), and 218 (GAPDH). PCR reactions were performed by using standard protocols, Platinum^tm^ Taq (Invitrogen) or TITANIUM^tm^ Taq (Clontech; Mountain View, CA) and an Eppendorf thermal cycler. PCR products were run on a 1.2% agarose gel to verify the predicted product sizes. PCR products were excised from the gels, extracted, purified (Qiaex II kit, Qiagen; Valencia, CA), and sequenced to verify the identity of the products.

Real-time PCR was performed using the StepOnePlus Real-Time PCR System (Applied Biosystems) according to the manufacturer’s instructions. Reactions were performed in triplicates, in 25 µl volumes with 0.5 µM primers and MgCl_2_ concentration optimized between 2 and 5 mM. Nucleotides, TaqDNA polymerase, and buffer were included in the SYBR Green PCR Master Mix (Applied Biosystems). A typical protocol designed on StepOne Software v2.0 (Applied Biosystems) included a holding stage for 10 min at 95°C and then a 15 s denaturation step, followed by 40 cycles with a 95°C denaturation for 15 s and 60°C annealing for 1 min. The melt curve stage included 95°C for 15 s, 60°C annealing for 1 min, and 95°C for 15 s. Measurements of the fluorescence were performed at the end of the 60°C annealing period. Ct values obtained from real-time PCR were normalized to GAPDH, and the fold difference between control and treated samples was determined using the ΔCt method and represented as a percentage change from baseline.

### Microscopy, Measurements and Cell Counts

Photomicrographs were obtained using a Leica DM5000B microscope equipped with epifluorescence and a Leica DC500 digital camera. Confocal images were obtained using a Zeiss LSM 510 imaging system at the Hunt-Curtis Imaging Facility at the Ohio State University. Images were optimized for color, brightness and contrast, multiple channels overlaid and figures constructed by using Adobe Photoshop^™^6.0. Cell counts were performed on representative images. To avoid the possibility of region-specific differences within the retina, cell counts were consistently made from the same region of retina for each data set.

Similar to previous reports [8], [16], [18], [19], immunofluorescence was quantified by using ImagePro 6.2 (Media Cybernetics, Bethesda, MD, USA). Identical illumination, microscope, and camera settings were used to obtain images for quantification. Retinal areas were sampled from 5.4 MP digital images. These areas were randomly sampled over the inner nuclear layer (INL) where the nuclei of the bipolar and amacrine neurons were observed. Measurements were made for regions containing pixels with intensity values of 68 or greater (0 = black and 255 = saturated); a threshold that included labeling in the bipolar or amacrine neurons. The total area was calculated for regions with pixel intensities >68. The average pixel intensity was calculated for all pixels within threshold regions. The density sum was calculated as the total of pixel values for all pixels within threshold regions. These calculations were determined for INL regions sampled from six different retinas for each experimental condition.
